# Tetracycline-Resistant *Vibrio cholerae* O1, Kolkata, India

**DOI:** 10.3201/eid1703.101176

**Published:** 2011-03

**Authors:** Koel Bhattacharya, Suman Kanungo, Dipika Sur, Banwari Lal Sarkar, Byomkesh Manna, Anna Lena Lopez, Manjira Bhattacharya, Suman Nandy, Swapan Kumar Niyogi

**Affiliations:** Author affiliations: National institute of Cholera and Enteric Diseases, Kolkata, India (K. Bhattacharya, S. Kanungo, D. Sur, B.L. Sarkar, B. Manna, M. Bhattacharya, S. Nandy, S.K. Niyogi);; International Vaccine Institute, Seoul, South Korea (A.L. Lopez)

**Keywords:** Resistance, tetracycline, antimicrobial resistance, Vibrio cholerae O1, bacteria, Kolkata, India, letter

**To the Editor:** Cholera, caused by toxigenic strains of *Vibrio cholerae* O1 or O139, continues to be a major cause of illness and death, particularly in developing countries. Treatment consists of early administration of rehydration therapy with appropriate oral or intravenous fluids. The World Health Organization recommends antimicrobial drug treatment for severely dehydrated patients with suspected cholera because it substantially shortens the duration of diarrhea by reducing the volume of watery stools, decreases fluid requirements, and limits transmission by decreasing fecal excretion of *V. cholerae* (*1*). The progressive increase in resistance to multiple drugs among strains causing clinical cases of cholera in developing countries is becoming a serious concern. We report the emergence of tetracycline-resistant *V. cholerae* O1 in a well-defined population in Kolkata, India, during 2007–2009.

During a 6-year surveillance period (2004–2009), we conducted a prospective, community-based study at an impoverished urban site in Kolkata. The goals of the study were to estimate the prevalence of cholera, describe its epidemiology, and identify potential risk factors that could be addressed by public health strategies.

Rectal swabs samples from patient with diarrhea were obtained, placed in Cary-Blair transport medium, and transported to the laboratory where they were processed for isolation and identification of *Vibrio* spp. Specimens were plated directly onto thiosulphate citrate bile salt sucrose agar (Eiken Chemical Company, Tokyo, Japan). The specimens were plated directly onto thiosulfate citrate bile salt sucrose agar. They were then incubated in alkaline peptone water (pH 8.6) for 6–8 h at 37°C and then plated onto the agar. After overnight incubation at 37°C, suspected colonies were tested biochemically and confirmed by slide agglutination with polyvalent O1 and monovalent Ogawa and Inaba antiserum (Difco Laboratories, Detroit, MI, USA).

Antimicrobial drug susceptibility testing was performed by using the disk diffusion technique on Mueller-Hinton agar (Difco Laboratories) with commercial disks (Oxoid, Cambridge, UK) and appropriate control strains (*2*). The MIC of tetracycline was determined with 101 randomly selected strains by Etest (AB Biodisk, Solna, Sweden) following manufacturer’s instructions.

During the 2004–2009 surveillance period, we isolated 809 *V. cholerae* O1 organisms, among which 624 (77%) were Ogawa and 185 (23%) were Inaba serotypes. The latter became the predominant serotype only in 2006. In 2007, a sudden upsurge in tetracycline resistance was noted among *V. cholerae* isolates, from 1% in 2004 to 76% in 2007 before decreasing to ≈50% in 2009. An increase in resistance to furazolidone and trimethoprim/sulfamethoxazole was also observed during the same period. Of the strains that were resistant to tetracycline, 99% were also resistant to furazolidone and trimethoprim/sulfamethoxazole (online Appendix Figure, www.cdc.gov/EID/content/17/3/568-appF.htm).

Among the tetracycline-resistant isolates (101 randomly selected strains), 43% had high-level resistance (MIC >16 µg/mL). In addition, 57% of *V. cholerae* O1 organisms had reduced susceptibility (i.e., MICs ranged from 8 µg/mL to 16 µg/mL).

Tetracycline is the drug of choice for treating cholera (*1*); however, during the 6-year period, we observed the emergence of tetracycline resistance among *V. cholerae* O1 isolates and a sudden upsurge in such resistance in 2007 when 76% of the isolates were resistant. Tetracycline resistance was also reported by Mhalu et al. (*3*), from an epidemic of cholera in Tanzania, where 76% of isolates were found to be resistant after 5 months of extensive use of this drug for treatment and prophylaxis. In a similar situation, the extensive prophylactic use of tetracycline triggered the rapid emergence and spread of tetracycline-resistant strains in Madagascar (*4*). Tetracycline is not used for prophylaxis in Kolkata, a known cholera-endemic area. Nonetheless, the emergence of resistant strains in our study area is not surprising because similar tetracycline-resistant strains of *V. cholerae* have been reported in Bangladesh (*5*), in Mozambique (*6*), and in another study of Kolkata (*7*). Notably, tetracycline-resistant *V. cholerae* O1 strains have also been responsible for major epidemics in Latin America, Tanzania, Bangladesh, and Zaire (*8*).

In our study, resistance to tetracycline among *V. cholerae* O1 isolates was <10% during 2004–2006. The reasons for the sudden rise of resistant strains in 2007 and their continued persistence are still unclear. Detailed molecular studies are underway to find the explanation. Alternative drugs, such as the newer fluoroquinolones, possess excellent activity against *V. cholerae* O1 and O139 serogroups. However, increased resistance to newer fluoroquinolones, such as ciprofloxacin and norfloxacin, among *V. cholerae* strains belonging to O1 serogroup has also been reported (*9*).

Resistance to commonly used antimicrobial drugs represents a critical public health problem because it complicates treatment and may result in longer hospital stays for patients. In addition, most of the population in developing countries cannot afford the newer and more expensive drugs. Our findings emphasize the need for continued surveillance of antimicrobial drug susceptibility patterns of *V. cholerae*. Providing early information on antimicrobial drug susceptibility to practitioners in affected areas will lessen the illness and death from this devastating disease.

## Figures and Tables

**Appendix:**
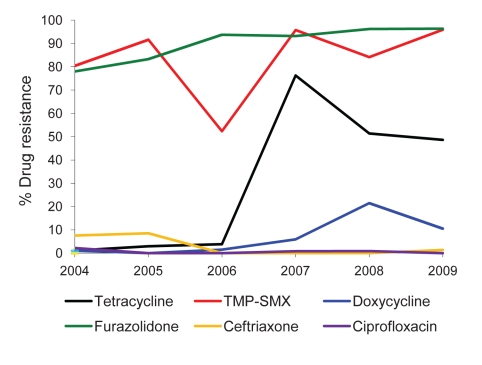
Figure**.** Antimicrobial drug resistance patterns of *Vibrio cholerae* O1, Kolkata, India, 2004–2009.
